# Costunolide Induces Apoptosis via the Reactive Oxygen Species and Protein Kinase B Pathway in Oral Cancer Cells

**DOI:** 10.3390/ijms22147509

**Published:** 2021-07-13

**Authors:** Hai Huang, Jun-Koo Yi, Su-Geun Lim, Sijun Park, Haibo Zhang, Eungyung Kim, Soyoung Jang, Mee-Hyun Lee, Kangdong Liu, Ki-Rim Kim, Eun-Kyong Kim, Youngkyun Lee, Sung-Hyun Kim, Zae-Young Ryoo, Myoung Ok Kim

**Affiliations:** 1 Department of Animal Science and Biotechnology, ITRD, Kyungpook National University, Sangju 37224, Korea; huanghai1227@126.com (H.H.); zhanghaibo615@163.com (H.Z.); wjddn5460@naver.com (E.K.); 2 Gyeongbuk Livestock Research Institute, Yeongju 36052, Korea; 79lee38@korea.kr; 3 School of Life Science, Kyungpook National University, Daegu 41566, Korea; sugeun624@hanmail.net (S.-G.L.); mooook15@naver.com (S.P.); wkdthdud21@naver.com (S.J.); 4 College of Korean Medicine, Dongshin University, Naju 58245, Korea; mhlee@dsu.ac.kr; 5 The Pathophysiology Department, School of Basic Medical Sciences, Zhengzhou University, Zhengzhou 450008, China; kdliu@zzu.edu.cn; 6 Department of Dental Hygiene, Kyungpook National University, Sangju 37224, Korea; rim0804@knu.ac.kr (K.-R.K.); ekkim99@knu.ac.kr (E.-K.K.); 7 Department of Biochemistry, School of Dentistry, Kyungpook National University, Daegu 41566, Korea; ylee@knu.ac.kr; 8 Department of Bio-Medical Analysis, Korea Polytechnic College, Chungnam 34134, Korea; shkim92@kopo.ac.kr

**Keywords:** costunolide, ROS, AKT pathway, apoptosis, oral cancer

## Abstract

Oral cancer (OC) has been attracted research attention in recent years as result of its high morbidity and mortality. Costunolide (CTD) possesses potential anticancer and bioactive abilities that have been confirmed in several types of cancers. However, its effects on oral cancer remain unclear. This study investigated the potential anticancer ability and underlying mechanisms of CTD in OC in vivo and in vitro. Cell viability and anchorage-independent colony formation assays were performed to examine the antigrowth effects of CTD on OC cells; assessments for migration and invasion of OC cells were conducted by transwell; Cell cycle and apoptosis were investigated by flow cytometry and verified by immunoblotting. The results revealed that CTD suppressed the proliferation, migration and invasion of oral cancer cells effectively and induced cell cycle arrest and apoptosis; regarding the mechanism, CTD bound to AKT directly by binding assay and repressed AKT activities through kinase assay, which thereby downregulating the downstream of AKT. Furthermore, CTD remarkably promotes the generation of reactive oxygen species by flow cytometry assay, leading to cell apoptosis. Notably, CTD strongly suppresses cell-derived xenograft OC tumor growth in an in vivo mouse model. In conclusion, our results suggested that costunolide might prevent progression of OC and promise to be a novel AKT inhibitor.

## 1. Introduction

Oral cancer is a type of head and neck squamous carcinoma (HNSC) and the sixth most common cancer worldwide with a high mortality rate [1]. More than 90% of oral cancer cases are of the oral squamous cell carcinoma (OSCC) type, which have been associated with alcohol consumption, human papillomavirus (HPV), smoking and betel quid chewing, and are the potential risk factors in the development of OSCC [2,3,4]. Unfortunately, delayed diagnosis is identified as one of the primary reasons [5] and most patients are diagnosed with advanced-stage disease when first discovered, leading to low rates of 5-year survival. Therefore, early detection and prevention play a vital role in controlling the burden of oral cancer. Currently used therapies for oral cancer include surgery, radiation therapy and chemotherapy alone or their combination, such as anlotinib [6], cetuximab [7] and PD-1/PD-L1 [8]. Cisplatin and 5-fluorouracil are commonly used in combination, and cisplatin is effective when used alone as well [9], however, the survival rate remains poor. Therefore, to improve the survival rate of patients with OSCC and the anticancer efficacy of drug therapy while reducing the toxicity and side effects of drugs, namely, the targeting of anticancer therapy has still been pursued for basic antitumor and clinical research.

The protein kinase B (PKB), also named AKT serine/threonine kinase, is an oncogenic protein. The AKT/mTOR pathway is one of the classical pathways involved in cell development processes such as cell growth, migration and survival in both healthy and tumor tissues. The Cancer Genome Atlas (TCGA) and previous studies have shown that the majority of HNCs possess alterations and are activated in the PI3K/AKT/mTOR pathway [10], which is also involved in physiological metabolism signaling, chemoresistance and radioresistance in oral cancer. Hence, targeting the AKT/mTOR pathway is an effective approach for treating patients with oral cancer, which has also been supported by previous studies [11,12]. An increasing number of studies have indicated that this pathway influences the progression of cell cycle by interfering with important cell cycle-related proteins, including cyclins, cyclin-dependent kinases and cyclin-dependent kinase inhibitors [13,14,15]. Some inhibitors based on this pathway are known to have a positive effect in various cancers; however, overcoming the emergence of drug resistance and side effects remains a huge challenge. Moreover, the increasing incidence of oral cancer necessitates the formulation of more efficient inhibitors.

Cell death is a crucial event for cancer cells under drug treatment, which is essential for maintaining tissue constancy by scavenging genetically compromised cells, including apoptosis and autophagy. Reactive oxygen species (ROS), a byproduct of normal cellular metabolism, plays a crucial role in intracellular signal transduction, cell growth and apoptosis [16]. Moreover, ROS levels are increased in cancer cells that could be more susceptible to oxidative stress, resulting in cell death. 

Which, therefore, can be exploited for cancer therapy. Furthermore, increasing levels of intracellular ROS could activate tumorigenesis-associated pathways such as MAPK and PI3K/AKT pathways [16,17]. In particular, the PI3K/AKT/mTOR pathway can be frequently activated by ROS through the inhibition of PTEN expression. Meanwhile, it can also promote ROS production to the extent by inhibiting the transcription factors FOXOs [17]. Therefore, the imbalance of ROS leads to increased oxidative stress in cancer cells, which thereby regulating certain signaling pathways. In addition, improving cancer therapies based on the toxic effects above a certain threshold level of ROS by inducing ROS accumulation directly or suppressing ROS scavenging systems represents a potential approach for eliminating cancer cells. In this context, some drugs have been considered to promote ROS generation, such as doxorubicin and motexafin gadolinium [18] and some preclinical drugs, including costunolide [19,20,21].

Natural products are considered as promising sources of cancer prevention and treatment drugs due to their ability to attack multiple molecular targets [22,23,24]. Costunolide (CTD) exhibits antitumor effects in various cancers due to its powerful activation, such as esophageal and colon cancers [21,25,26,27]. It exhibits various anticancer activities by reducing the oncogene expression, regulating cell cycle, inducing apoptosis, suppressing metastasis and modulating drug resistance [28,29]. However, the anticancer activity of costunolide in oral cancers still remains unclear and requires further confirmation. Therefore, this study aimed to investigate the potential of costunolide in inhibiting oral cancer cell proliferation, migration and invasion and inducing cell apoptosis and cell cycle arrest in vitro and to identify the molecular mechanisms underlying its anticancer activity. In addition, the antiproliferative mechanism of costunolide against oral cancer was further examined by evaluating ROS production and related expression of caspase proteins. Eventually, we considered that costunolide suppressed oral cancer growth through the ROS and AKT pathway.

## 2. Results

### 2.1. AKT Expresses in OSCC Patients Tissue

**Figure 1:**
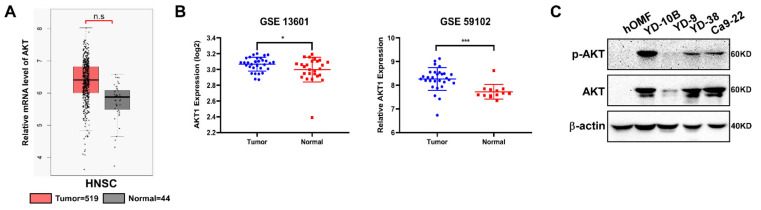
AKT expressed in OSCC. (**A**) The average expression level of AKT in patients with HNSC in TCGA and GTEx oral cancer dataset; (**B**) AKT expression in OSCC from GSE13601 and GSE59102. * *p* < 0.05, *** *p* < 0.001 indicate a significant difference compared with control; (**C**) The expression of AKT in oral cancer cell lines.

AKT (AKT1) is expressed in various cancers. To determine the role of AKT in HNSC, we first evaluated AKT expression in HNSC at the mRNA level and observed no significant differences between the tumor and normal group (data obtained from http://gepia.cancer-pku.cn/, accessed on 5 February 2021) (Figure 1A). However, we observed that AKT expression was significantly (*p* < 0.05) increased in tumor tissues compared to that in normal adjacent tissues, as analyzed from the dataset of GSE13601 and GSE59012 (Figure 1B). The results suggested that the mRNA level of AKT is not significantly changed in HNSC patients but is significantly altered in OSCC patients. In addition, we also examined the AKT expression in oral cancer cell lines, which revealed that AKT is more expressed in YD-10B, YD-38 and Ca9-22 than in YD-9 (Figure 1C).

### 2.2. The Target of Costunolide Is AKT

**Figure 2:**
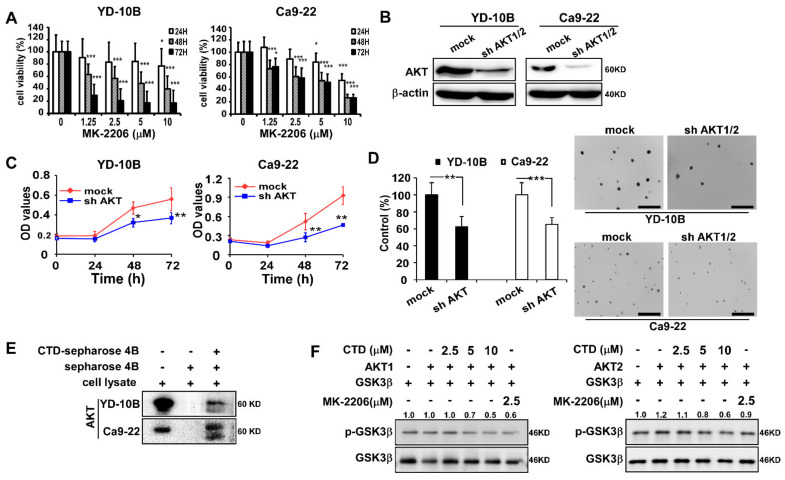
AKT is a target of Costunolide. (**A**) The proliferation of OC cells under the AKT inhibitor (MK-2206) was determined by the MTT assay at 24, 48 and 72 h. (**B**) AKT expression was detected by the western blot assay after knockdown of AKT in oral cancer cells. (**C**) Cell growth was examined in oral cancer cells after knockdown of AKT by the CCK-8 assay. (**D**) Anchorage-independent growth was evaluated in oral cancer cells after knockdown of AKT (Scale bar = 200 μm). Data are represented as mean ± SD of values from triplicate samples. * *p* < 0.05, ** *p* < 0.01 and *** *p* < 0.001 indicate a significant difference compared with control. (**E**) Costunolide binds with AKT. (**F**) The AKT kinase activity was inhibited by costunolide and downregulated the phosphorylation of GSK3β, the AKT substrate.

Next, we evaluated the effects of treating oral cancer cells with an AKT inhibitor or by knockdown of AKT. Results showed that treatment with MK-2206 (AKT inhibitor) [15,30] effectively inhibited the proliferation of YD-10B and Ca9-22 cells (Figure 2A). Immunoblot analysis revealed that the AKT expression was decreased by sh-AKT compared with the sh-mock treatment (Figure 2B). Moreover, the elimination of AKT1/2 decreased the growth of oral cancer cells compared with the mock control (Figure 2C). The number of colonies was considerably decreased in the anchorage-independent cell growth assay after AKT knockdown (Figure 2D).

Our previous results showed that AKT is a target of CTD, which was confirmed not only by a binding assay but also by a computational model and site-directed mutagenesis [31]. Therefore, we determined the interaction between CTD and AKT in oral cancer cells using the pull-down assay and again confirmed the binding between CTD and AKT (Figure 2E). For further determining the effect of CTD on AKT, we conducted an in vitro kinase assay with active AKT in the presence of CTD at different doses and observed that p-GSK3β (one of AKT substrates) was inhibited dose-dependently under the CTD treatment (Figure 2F). These results again suggested that CTD is a potential effective inhibitor of AKT.

### 2.3. The Effect of Costunolide on the Viability of Oral Cancer Cells

**Figure 3:**
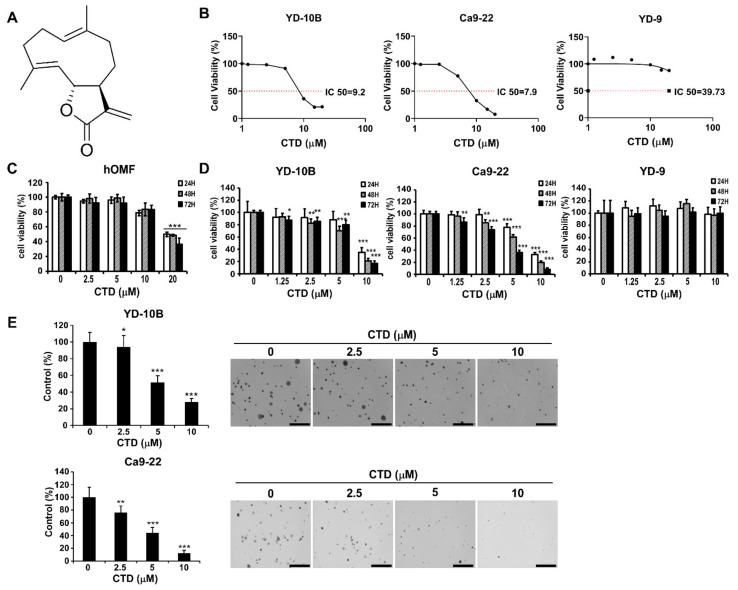
Costunolide inhibits proliferation of oral cancer cells. (**A**) The structure of Costunolide. (**B**) The IC50 value of oral cancer cells (YD-10B, Ca9-22 and YD-9) was examined by MTT assay at 24 h. (**C**) The effects of CTD on the growth of healthy oral epithelial cells. (**D**) The effects of CTD on the growth of YD-10B, Ca9-22 and YD-9 oral cancer cells were determined by the MTT assay at 24, 48 and 72 h. (**E**) The effects of CTD on the colony formation of oral cancer cells were evaluated by the soft agar assay and representative photographs of colony formation are shown (100×). Data are represented as mean ± SD of values from triplicate samples; * *p* < 0.05, ** *p* < 0.01 and *** *p* < 0.001 indicate a significant difference compared with control.

Persistent proliferation is a feature of cancer cells. To examine the effect of CTD (structure as shown in Figure 3A) on the proliferation of oral cancer cells, we treated them with different concentrations of CTD (0–20 μM) for 24 h and then determined the percentage of viable cells by the MTT assay. As shown in Figure 3B, the effective doses of CTD that inhibited 50% growth (IC50) in YD-10B, Ca9-22 and YD-9 are 9.2, 7.9, and 39.6 μM, respectively. Additionally, the result of the in vitro toxicity assay of CTD on healthy oral epithelial cell (hOMF) showed that CTD began to significantly reduce cellular viability of normal cells at a concentration of 20 μM (Figure 3C). Through detailed statistical analysis, we selected YD-10B and Ca9-22 as our target cells and 10 μM as the concentration of CTD for future experiments. The MTT assay results also showed that CTD dose-dependently inhibited cell growth effectively (Figure 3D). Moreover, in the anchorage-independent cell growth assay, it was observed that CTD remarkably suppressed the colony formation of oral cancer cells dose-dependently (Figure 3E). 

### 2.4. Costunolide Efficiently Inhibits Cell Migration and Invasion

**Figure 4:**
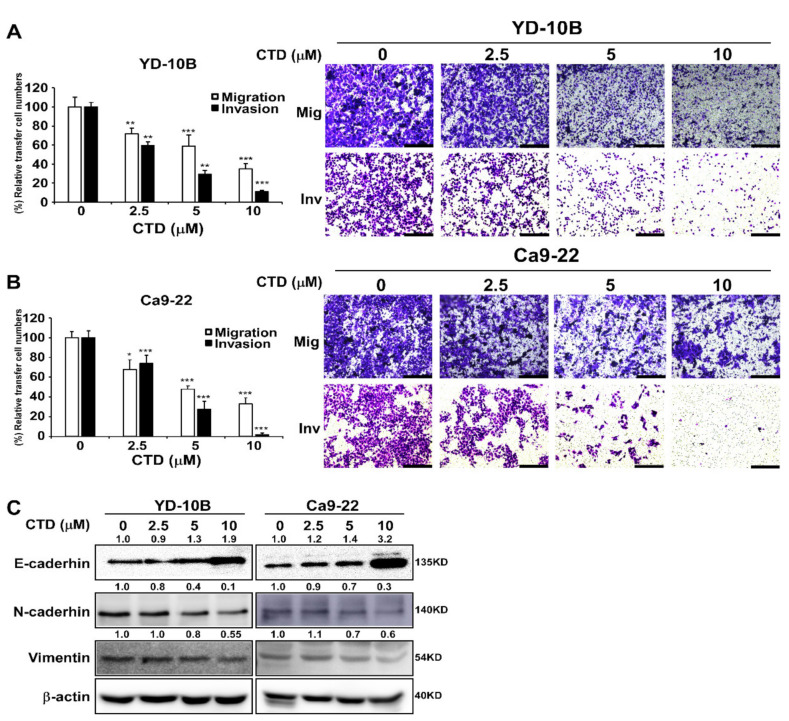
Costunolide inhibits the migration and invasion of oral cancer cells. (**A**) The migration and invasion of YD-10B cells was examined by the transwell membrane with or without the Matrigel assay after treatment with different doses of CTD at 24 h and representative photographs (Scale bar = 200 μm). (**B**) The migration and invasion of Ca9-22 cells was determined by the transwell membrane with or without the Matrigel assay after treatment with different doses of CTD at 24 h and representative photographs (Scale bar = 200 μm). Data are expressed as mean ± SD of values from triplicate samples. * *p* < 0.05, ** *p* < 0.01 and *** *p* < 0.001 indicate a significant difference compared with control. (**C**) The expression levels of E-cadherin, N-cadherin and vimentin were detected by western blotting after treatment with costunolide.

Migration and invasion play a vital role in the initial progression of cancer that facilitates metastasis [32]. Therefore, to determine the potential inhibitory effect of CTD on the migration and invasion of oral cancer cells, we conducted transwell assays with uncoated (for migration) or Matrigel-coated (for invasion) filters and observed that CTD significantly inhibited the migration and invasion of oral cancer cells in a dose-dependent manner after a 24-h treatment (Figure 4A,B). Moreover, the expression of E-cadherin was increased and the expressions of N-cadherin and vimentin were decreased under the CTD treatment, supporting that the migration and invasion of cancer cells was suppressed (Figure 4C). These results demonstrated that CTD has the potential to inhibit the migration and invasion of oral cancer cells.

### 2.5. Costunolide Induces Cell Cycle Arrest in the G2/M Phase

**Figure 5:**
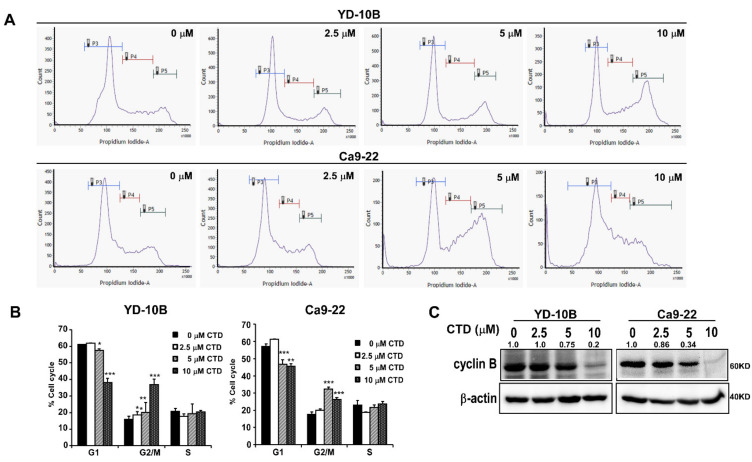
Costunolide induces cell cycle arrest in the G2/M phase of oral cancer cells. (**A**) Analysis of results from the cell cycle assay. Cells were treated with 0, 2.5, 5 or 10 μM CTD and then incubated for 24 h. Data are shown as mean ± SD of values from triplicate samples. * *p* < 0.05, ** *p* < 0.01 and *** *p* < 0.001 indicate a significant difference compared with control. (**B**) Image indicating cell cycle distribution. (**C**) Expression of cell cycle markers in oral cancer cell lines.

To confirm whether the antiproliferative activity of costunolide in oral cancer corresponds to the regulation of cell cycle, we conducted flow cytometry to examine cell cycle progression after treatment with the indicated concentrations of CTD (0–10 μM). CTD treatment resulted in an increase in the number of G2/M phase cells, along with a decrease in the number of G1 phase cells, in two cell lines as shown in Figure 5A,B, which was supported by the decreased expression of cyclin B in a dose-dependent manner (Figure 5C). This suggested the occurrence of a cell cycle arrest in the G2/M phase after the CTD treatment.

### 2.6. Costunolide Triggers Apoptosis of Oral Cancer Cells

**Figure 6:**
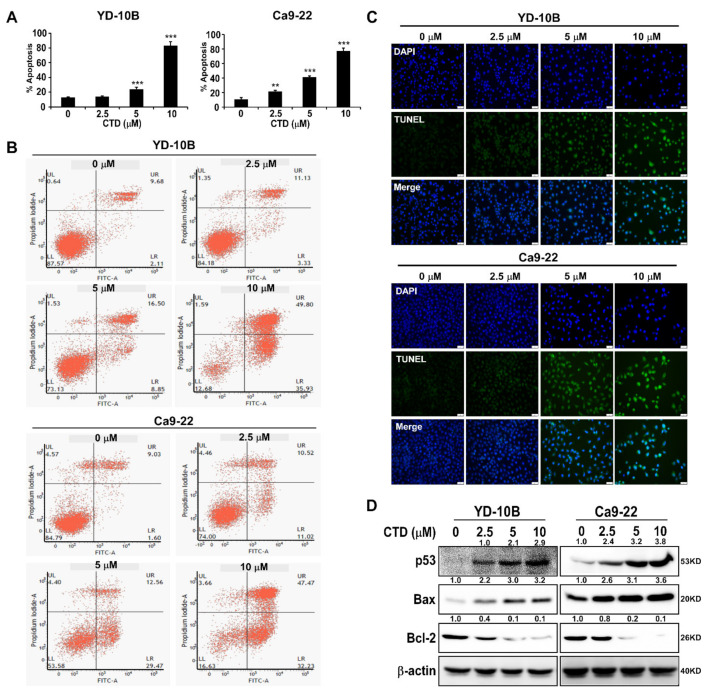
Costunolide triggers apoptosis of oral cancer cells. (**A**) Analysis of the results of apoptosis assay. Data are expressed as mean ± SD of values from triplicate samples. ** *p* < 0.01 and *** *p* < 0.001 indicate a significant difference compared with control. (**B**) Image of apoptotic cell populations. (**C**) The apoptosis of indicated cells treated with or without CTD at different concentrations (0, 2.5, 5 or 10 μM) was measured by TUNEL assay (Scale bar = 200 μm). (**D**) Expression of apoptotic markers in oral cancer cell lines. Cells were treated with 0, 2.5, 5 or 10 μM CTD and then incubated for 24 h.

Apoptosis is one of the negative growth factors in cell growth after treatment with drugs. Therefore, we analyzed the oral cancer cells by costaining with Annexin V/PI after the CTD challenge and observed that the CTD treatment triggered apoptosis in oral cancer cells (Figure 6A,B). We also observed the apoptosis of cells treated with CTD at different concentrations using the TUNEL assay. Results showed that the CTD treatment significantly and dose-dependently promoted the apoptosis of oral cancer cells compared with the vehicle group (Figure 6C). Meanwhile, the CTD treatment increased the up-regulation of the p53 and Bax and decreased the expression of Bcl-2 in a dose-dependent manner (Figure 6D), which confirmed this result. Therefore, these results indicated that the CTD treatment triggered cell apoptosis in a dose-dependent manner.

### 2.7. ROS Induction in Costunolide-Treated Oral Cancer Cells

**Figure 7:**
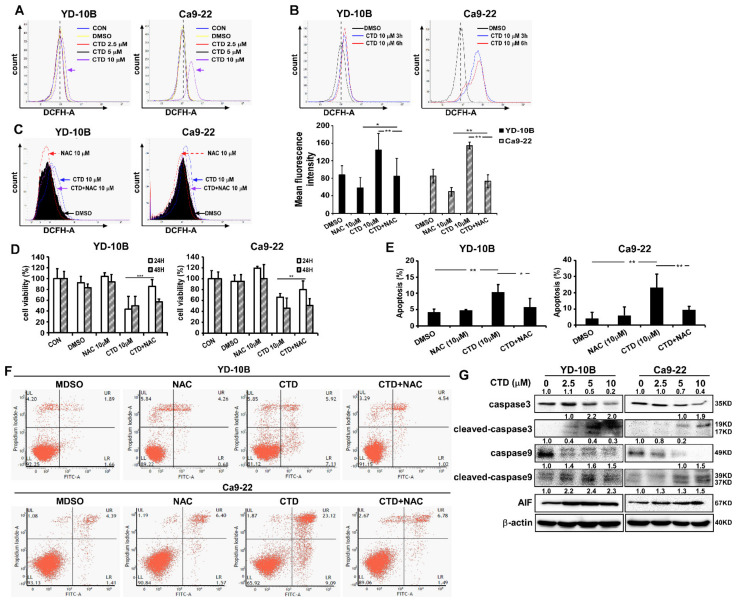
Induction of ROS generation by costunolide was examined in human oral cancer cells. (**A**) Flow cytometric analysis of ROS generation after the CTD treatment at different doses. (**B**) Analysis of ROS production after treatment with 10 μM CTD at different time points. (**C**) Cells were treated with 10 μM CTD and 10 mM NAC each alone or in combination for 24 h. DCFH-A staining was used to evaluate ROS production (left). The mean fluorescence intensity of DCFH-DA staining is shown in oral cancer cells (right). (**D**) Cell proliferation was detected by the MTT assay in oral cancer cells treated with 10 μM CTD and 10 mM NAC each alone or in combination at different time points. (**E**) Cells treated with 10 μM CTD and 10 mM NAC each alone or in combination for 24 h; cell apoptosis was examined by the flow cytometry assay. (**F**) Image of apoptotic cell populations. (**G**) ROS production was induced after the CTD treatment leading to some protein expression. Values are expressed as mean ± SD (*n* = 3); * *p* < 0.05, ** *p* < 0.01 and *** *p* < 0.001 indicate a significant difference compared with control.

Previous studies have demonstrated that ROS production is an essential effect of CTD during cell death in several types of cancers [25,33]. Therefore, to investigate whether CTD inhibited oral cancer cell growth through ROS production, we treated the two oral cancer cell lines with various doses of CTD and selected 10 μM for different time periods. Results showed that CTD rapidly and dramatically enhanced ROS generation in oral cancer cells (Figure 7A,B). When cells were pretreated with NAC (an ROS inhibitor) at 10 mM for 2 h, the ROS production was decreased compared to that with CTD alone or DMSO control (Figure 7C). Furthermore, we combined NAC with CTD to confirm the role of CTD in inhibiting the proliferation of oral cancer cells through ROS generation (Figure 7D). As anticipated, NAC significantly decreased the CTD-induced ROS production and reversed the CTD-induced apoptosis (Figure 7E,F). We next analyzed the caspase activity by western blotting and observed that the expressions of cleaved caspase-3 and caspase-9 were promoted and the expression of AIF (Apoptosis Inducing Factor) was increased related to ER stress (endoplasmic reticulum stress) (Figure 7G). These findings suggested that CTD triggered apoptosis in association with excess ROS generation.

### 2.8. Costunolide Inhibits Oral Cancer Cell Growth through the AKT Pathway

**Figure 8:**
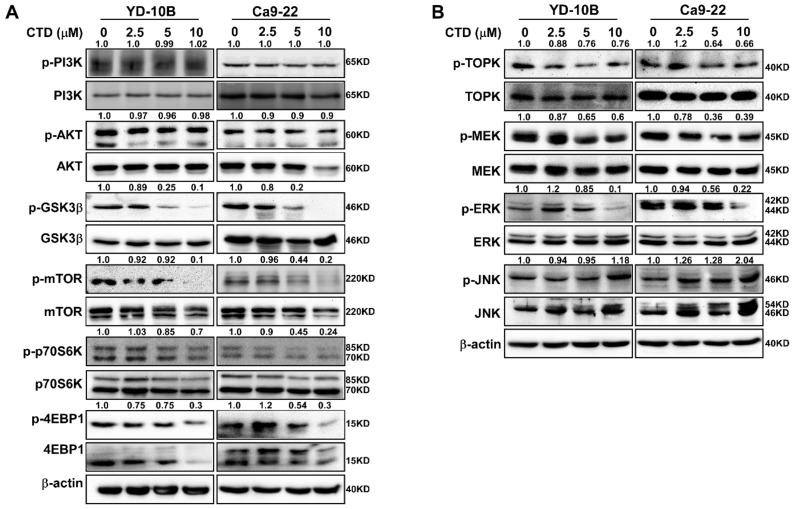
Costunolide suppresses AKT signaling in oral cancer cells. Cells were treated with DMSO or 2.5, 5 or 10 μM CTD for 24 h. Proteins were extracted, (**A**) the expression levels of AKT and proteins related to AKT/mTOR pathway; (**B**) the expression levels of TOPK and some proteins involved in MAPK pathway, β-Actin was used as a loading control.

The AKT/mTOR axis is one of the most reported signaling pathways in oral cancer [11,12]. Therefore, to elucidate the role of the AKT/mTOR pathway in the molecular mechanism of CTD treatment underlying cell proliferation and apoptosis, we analyzed the expression of proteins that participate in the AKT pathway. Results showed that CTD significantly suppressed the phosphorylation of mTOR and GSK-3β dose-dependently (Figure 8A). In particular, the form of phosphorylation of AKT and total AKT were not different in oral cancer cells, especially in YD-10B. Meanwhile, we also observed that Ca9-22 cells were more sensitive than the YD-10B cells when treated with CTD, which led to a slightly decreased formation of AKT at high concentrations. Moreover, TOPK is another substrate of AKT that plays an important role in cell proliferation and metastasis. Therefore, we investigated the TOPK expression and its related proteins. As depicted in Figure 8B, the phosphorylations of MEK and ERK are also reduced under CTD treatment. However, the phosphorylation of JNK was increased under the same CTD treatment condition, suggesting that the ROS/JNK pathway was activated.

### 2.9. Costunolide Inhibits Tumor Growth of Cell-Derived-Xenograft (CDX)

**Figure 9:**
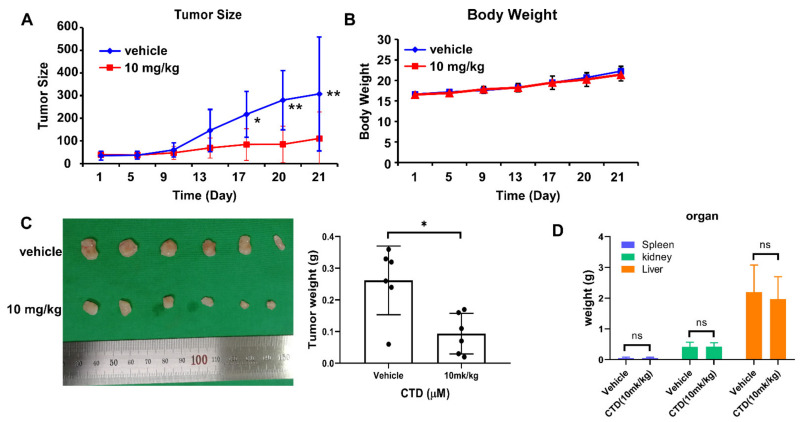
Costunolide inhibits tumor growth of cell-derived-xenograft. Mice were divided into 2 groups (n = 6) and treatment with vehicle and 10 mg/kg of CTD by intraperitoneally administered, respectively. (**A**) CTD inhibits oral cancer cell growth in vivo. (**B**) Body weight from treated or vehicle groups of mice were obtained every 4 days and showed that CTD has no effect on body weight. (**C**) Tumor weight decreased after CTD treatment compared to vehicle treatment, (Left: Representative photographs of tumors). (**D**) Weight of Liver, spleen and kidney in between vehicle control and CTD-treated groups in CDX experiment. No significance was observed between control and experiment groups. All data are shown as mean values ± S.D. * *p* < 0.05, ** *p* < 0.01 indicate a significant difference compared with control; *ns* represent no significant difference.

To investigate the anti-tumor role of CTD on oral cancer progression in vivo, we subcutaneously injected nude mice with Ca9-22 cells and then treated them with vehicle or 10mg/kg CTD for 21 days, respectively. Results indicated that CTD significantly reduced Ca9-22 CDX tumor growth. No obvious difference in body weight between the control and experimental groups was observed (Figure 9A–C). Mice were then euthanized, and the liver, spleen and kidney were collected and weighed. The weight of the liver, spleen and kidney indicated no significant toxicity of CTD to mice at the administered concentrations (Figure 9D). The results of the in vivo experiment suggest that CTD can inhibit oral cancer growth in vivo by modulating AKT activity.

## 3. Discussion

Currently, the therapy and diagnosis of oral cancer have attracted extensive research attention due to its high rates of metastasis and misdiagnosis. Despite the development of novel therapies such as targeted therapy, gene therapy and FDA-approved clinical drugs, the treatment of patients with oral cancer still faces serious challenges due to metastasis, drug resistance and lack of response. Therefore, further exploration of chemotherapeutic agents is imperative. Considering the hyperactivation of the PI3K/AKT/mTOR pathway in Head and Neck squamous cell carcinoma (HNSCC), targeting this pathway has become a significant approach for oral cancer treatment, where are responsible for in both in therapy resistance and cell proliferation to some extent [11,12,34]. Till date, several small molecule inhibitors of the PI3K/AKT/mTOR pathway have been investigated in preclinical studies; however, only a few inhibitors are currently approved for clinical treatment of human cancers [35]. Moreover, the application of AKT, mTOR and PI3K inhibitors to patients with HNCS has been achieved only up to phase II of clinical trials, and some studies have even investigated them in combination with cetuximab or cytostatic drugs for oral cancer therapy [12]. This feature has led to the drug development of these compounds, and in particular, AKT inhibitors have been demonstrated to promote apoptosis in combination with currently used anticancer drugs.

The anti-cancer effects of costunolide (CTD), such as induction of apoptosis, inhibition of cell proliferation in vitro and suppression of angiogenesis and metastasis, have been documented in several studies. However, the function of CTD in oral cancer has not been fully characterized. Therefore, in the present study, we investigated the function of CTD using oral cancer cell lines. Previous studies revealed that CTD suppressed cancer cell proliferation by altering cell cycle progression through the regulation of cyclins and cyclin-dependent kinases (CDK) [33,36,37]. Additional literature has also reported that CTD can induce apoptosis via activated p53 or mitochondrial damage through increased production of reactive oxygen species (ROS) in colon, gastric and lung cancers [21,37,38,39]. These observations suggest that CTD-induced ROS generation played a critical role in this process, which is consistent with our results. Moreover, CTD represses hepatic fibrosis through the WW domain-containing protein-2-mediated Notch3 degradation and enhances the sensitivity of doxorubicin treatment to chronic myeloid leukemia cells through the PI3K/AKT pathway [40,41]. Our previous study also confirmed that CTD inhibited cell growth and triggered apoptosis through the MDM2/P53 pathway by targeting AKT in colon cancer [42]. Obviously, CTD is a potential inhibitor in cancer therapy.

In current study, our findings revealed that treating oral cancer cells with CTD resulted in significant inhibition of cell proliferation, anchorage-independent colony formation and induction of G2 phase cell cycle arrest and apoptosis (Figure 3, Figure 5 and Figure 6). The expression of p53 and bax was increased and that of Bcl-2 was reduced, supporting the antitumor role of CTD in oral cancer cells. Our findings also revealed that the migration and invasion of cells were suppressed under CTD treatment (Figure 4), which was contributed by the increased expression of E-cadherin and decreased expression of N-cadherin and vimentin. Furthermore, Alkhadar et al. (2020) reported that nerve growth factor induced metastasis, migration and invasion through the activation of the PI3K/AKT pathway in oral and salivary tumor cells [43], whereas the PI3K/AKT pathway is widely involved in OSCC. It is well known that this axis is associated with various hallmarks of cancer, including proliferation, survival and metastasis. We next confirmed whether AKT is a target of CTD in oral cancer, which directly binds with AKT (Figure 2E). We observed that knockdown of AKT in two cell lines reduced their growth and colony formation as assessed by the shRNA assay (Figure 2C,D). The findings revealed that CTD is a potential inhibitor of oral cancer cells, and AKT is a target of CTD. Regarding the mechanism, the kinase assay showed that CTD inhibited AKT kinase activity dose-dependently, thereby suppressing the phosphorylation of its substrates such as GSK3β (Figure 2F). Furthermore, we also draw the conclusion is that costunolide bind to the AKT1 and AKT2, and it inhibits AKT1 is better than AKT2. In addition, CTD treatment downregulated the expression of GSK3β and mTOR, as shown in Figure 8A. 

Natural compounds with their multi-target properties can inhibit the activation of PI3K/AKT and MAPK signaling pathways in the treatment of cancer [44]. Therefore, TOPK is both an AKT substrate and a protein that belongs to the MAPK pathway, which is particularly significant here. Our results further showed that CTD also decreased the MAPK pathway through TOPK inactivation (Figure 8B). 

ROS have been identified as important molecules in the regulation of cell survival or cancer cell death [16], which as the other mechanisms of CTD activity in cancer treatment.

Our findings showed that CTD induced significant ROS generation; however, pretreatment with NAC significantly rescued the CTD-associated cell proliferation and apoptosis, suggesting that CTD exerts apoptotic effects through ROS generation in oral cancer cells (Figure 7). Previous studies have reported that the ROS/MAPK pathways participate in inducing apoptosis in oral carcinoma and renal cell cancer, respectively [45,46], and our findings are consistent with this report. These results suggested that CTD induced apoptotic changes through the activation of the ROS/JNK pathway.

Tumor xenograft animal models including subcutaneous tumor xenograft model, ortho topic tumor xenograft model and patient-derived tumor xenograft model are vital tools for cancer study [47]. In this study, the tumor size and weight were decreased by treatment with CTD compared to vehicle control. Meanwhile, the body weight of mice maintained stability from whole process (Figure 9). Furthermore, without change in weight of liver or spleen and H&E staining compared to vehicle control. All which revealed that there is no toxicity of CTD at this dose. Therefore, CTD is an effective drug for oral cancer treatment.

**Figure 10:**
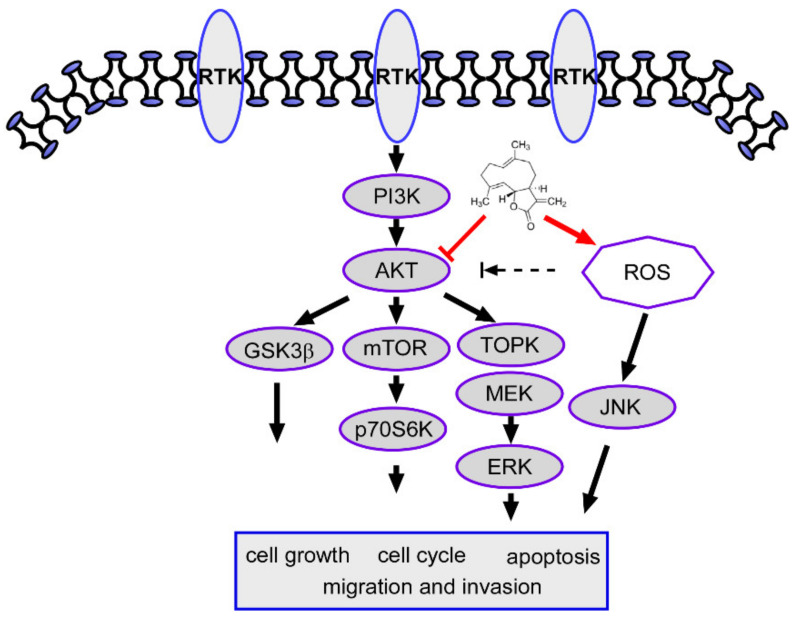
Costunolide inhibits oral cancer cell proliferation and induces apoptosis through the ROS and AKT signaling pathway.

Altogether, our study findings preliminarily demonstrate that CTD inhibits proliferation and induces apoptosis of human oral cancer cells by targeting AKT to downregulate the expression of GSK3β, mTOR and TOPK, thus inhibiting cell growth, migration and apoptosis. CTD can also induce apoptosis by accelerating ROS generation (Figure 10). In this study, we evaluated the efficacy of CTD in in vitro and in vivo study; the inhibition of oral cancer cell growth was obviously significant. Obviously, our study identified a target of CTD and illustrated a potential mechanism. These findings provide a foundation in cell biology for the development of CTD as a therapeutic drug for human oral cancer, which also first time to report CTD effect oral cancer via AKT pathway.

Through targeting AKT and blocking the signals of downstream targets such as mTOR, GSK3b and TOPK, cell cycle arrest and apoptosis are induced, and cell proliferation is also inhibited. Moreover, ROS generation was increased under CTD stimulation, thereby activating JNK expression to inhibit oral cancer cell growth.

## 4. Materials and Methods

### 4.1. Reagents

Costunolide (CAS: 553-21-9, purity ≥ 97%) was purchased from Jonk Biological Tech nology Co. Ltd. (Wuhan, China) and authenticated by high-performance liquid chromatography. Media (DMEM and MEM) were obtained from Gibco (Grand Island, NY, USA). CCK-8 assay reagents were acquired from Dojindo Molecular Technologies, Inc.,(Tokyo, JAPAN). MTT (3-(4,5-dimethylthiazol-2-yl)-2,5-diphenyltetrazolium bromide (Sigma). Antibodies to detect p-AKT, AKT, p-MEK, MEK, pERK, ERK, p-mTOR, mTOR, p-GSK3β, GSK3β, E-cadherin, N-cadherin and vimentin were purchased from Cell Signaling Technology, and antibodies to detect cyclin B1, p53, Bcl-2 and β-actin were obtained from Santa Cruz Biotechnology.

### 4.2. Cell Culture

Human Oral Squamous Cell Carcinoma (OSCC) cell lines YD-10B, YD-9 and Ca9-22 are derived from human tongue cancer, buccal mucosa and oral gingival cancer, respectively; and Normal oral epithelial cell (hOMF) lines were purchased from the Type Culture Collection of the Korea Cell Bank. Cells were grown cultured in DMEM supplemented with penicillin–streptomycin (100 U/mL) and 10% fetal bovine serum (Biological Industries, Kibbutz Beit-Haemek, Israel). All cells were maintained in a standard incubator with 5% CO_2_ at 37 °C. Cells were also cytogenetically tested and authenticated before being frozen. Each vial of frozen cells was thawed and maintained in culture for a maximum of eight generations.

### 4.3. Analysis of AKT Expression

The mRNA expression of AKT was evaluated in patients with oral cancer (data obtained from http://gepia.cancer-pku.cn/, accessed on 5 February 2021). Transcript data (expressed in TPM+1 units) was log2 transformed and plotted within the GEPIA website. The log2FC was defined as median (Tumor) __ median (Normal). Genes with higher |log2FC| values and lower q values than the default thresholds were considered differentially expressed genes. Furthermore, published microarray datasets (NCBI/GEO/GSE13601 and GSE59012) were queried and downloaded from the Gene Expression Omnibus. The AKT transcript expression data derived from normal and tumor groups was parsed and analyzed. Then statistics data (*t*-test) and results as marked log values. 

### 4.4. Cell Viability Assay

Cells (2–3 × 10^3^ cells/well) were seeded in 96-well plates, and after 24 h, they were treated with vehicle and different doses of CTD (2.5, 5 or 10 µM). Cell viability was determined by the MTT assay at 24, 48 and 72 h, respectively. For anchorage-independent cell growth assessment, cells (8 × 10^3^ per well) were suspended in media containing 10% FBS for cell maintenance. After that, 0.3% agar with various concentrations of CTD was filled into a top layer upon a base layer of 0.5% agar containing the same corresponding doses of CTD in 6-well plates; three replicate wells were set for each concentration of the compound. After two weeks, the colonies were photographed using a microscope and calculated using the Image-Pro Plus software (v.6.0) program (Media Cybernetics, Rockville, MD, USA).

### 4.5. Cell Cycle and Apoptosis Analysis

Cells (2 × 10^5^) were seeded in 60-mm plates, and after 24 h, they were treated with vehicle or different doses of CTD for another 24 h. Cells were fixed in 70% ethanol and stored at −20 °C overnight. This was followed by staining with propidium iodide for 15 min at room temperature (RT); For apoptosis assay, cells (2 × 10^5^) were seeded into 60-mm dishes and cultured at 37 °C in a 5% CO_2_ incubator. After treatment with CTD for 24 h, cells were harvested and stained with Annexin V and propidium iodide at RT for 15 min. The stained cells were analyzed for cell cycle and apoptosis using a BD FACS Calibur Flow Cytometer (BD Biosciences, San Jose, CA, USA). 

### 4.6. TUNEL Assay

Apoptotic cell death was evaluated by the TUNEL assay. Briefly, cells (1 × 10^5^) were seeded in a chamber slide and incubated in DMEM containing CTD for 24 h. The apoptotic cells were detected by the TUNEL assay using an in situ apoptosis detection kit (Takara, Shiga, Japan) according to the manufacturer’s instructions. The cells were stained with DAPI, mounted and then examined by fluorescence microscopy (Leica Microsystems, Germany).

### 4.7. Migration and Invasion Assays

For cell migration analysis, cells were directly seeded into 8-μM transwell filters (Corning, Corning, NY, USA). The major difference in cell invasion analysis is that Matrigel was added into the upper compartment of the transwell, and the remaining experimental protocol was similar to that of the migration assay. Briefly, cells (5 × 10^4^) suspended in a serum-free medium were seeded onto the upper compartment of the transwell, and the lower chambers were filled with medium containing 10% FBS and various concentrations of CTD. After 24 h, noninvading cells were removed and the migrating and invading cells were fixed with 4% paraformaldehyde, permeabilized with 100% methanol and then stained with 0.5% crystal violet for 10 min. Finally, the cells were photographed under a microscope (magnification, 100×) and counted using the Image-Pro Plus software (v.6.1) program (Media Cybernetics).

### 4.8. Measurement of Reactive Oxygen Species Generation 

Cells were plated at 2 × 10^5^ density in a 60-mm dish and allowed to attach for 12 h. They were then treated with CTD for another 12 h. To avoid the effect of ROS production by CTD, pretreatments with an ROS inhibitor (NAC, N-acetyl-L-cysteine) were conducted at 10 mM for 2 h. Subsequently, after treatment with CTD for 12 h, the cells were exposed to DCFH-DA (10 μM) at 37 °C for 30 min in the dark, collected and washed gently, and ROS production was evaluated by flow cytometry analysis (BD Biosciences, San Jose, CA, USA).

### 4.9. Western Blot Assay

Cells were lysed with a NP-40 lysis buffer (additional protease inhibitor cocktail, dephosphorylate inhibitor tablets and 1 mmol/L phenylmethylsulfonyl fluoride [PMSF] are added), and the protein concentration of each lysate was determined using the BCA Quantification Kit (Solarbio). The protein extracts (50 μg) were loaded onto 8–10% SDS-PAGE gels, transferred to polyvinylidene fluoride membranes and treated with a primary antibody at 4 °C overnight after blocking with 5% Bovine serum albumin (BSA) or nonfat dry milk in a 1× TBST. Next, the membranes were incubated with an HRP-conjugated secondary antibody for 1–2h at RT. Protein bands were visualized using the enhanced chemiluminescence (ECL) detection reagent (GE Healthcare Life Science).

### 4.10. AKT Kinase Analysis In Vitro

GSK3β was used as the substrate for an in vitro kinase assay using 200 ng of active AKT1 and AKT2. Reactions were conducted in a 1× kinase buffer (25 mM Tris–HCl pH 7.5, 5 mM β-glycerophosphate, 2 mM dithiothreitol [DTT], 0.1 mM Na3VO4, 10 mM MgCl2 and 5 mM MnCl2) containing 100 μM ATP at 30 °C for 30 min. Reactions were terminated by adding 2× protein-loading dye, and proteins were detected by western blotting.

### 4.11. Binding Assay

Oral cancer cell lysates (500 μg) were incubated overnight with costunolide-Sepharose 4B beads (or only Sepharose 4B beads) in a reaction buffer containing 50 mM Tris–HCl (pH 7.5) 5 mM EDTA, 150 mM NaCl, 1 mM dithiothreitol, 0.01% NP-40 and 2 mg/mL bovine serum albumin in a 4 °C rotator. After incubation under gentle shaking, the beads were washed three times with a washing buffer containing 50 mM Tris–HCl (pH 7.5), 5 mM EDTA, 150 mM NaCl, 1 mM dithiothreitol and 0.01% NP-40. Binding was visualized by western blotting with an AKT antibody.

### 4.12. Lentiviral Infection

The packaging and viral vectors (pMD2G, psPAX2 and shAKT1/2) were transfected into 293T cells using the FuGENNE HD Transfection Reagent (Promega). The viral particles were harvested by filtration using a 0.22-μm filter and then stored at −80 °C. The cultured oral cancer cells were infected with viral particles and 8 μg/mL polybrene (Millipore, Billerica, MA, USA) for 24 h. Then, the cells were selected with puromycin for another 24 h and the selected cells were used for subsequent experiments. Cells (1 × 10^3^/well) were seeded in 96-well plates, and after incubation for 24, 48 or 72 h, cell proliferation was detected by the CCK-8 assay described earlier in the cell viability assay.

### 4.13. In Vivo Analysis

Six-week-old nude mice were obtained from Charles River. The mice were maintained in a specialized environment for 1 week prior to conducting in vivo experiments. The animal experiments study was approved by the Ethics Research Committee of Kyungpook National University (Dae-gu, South Korea). Ca9-22 cells (1 × 10^7^ in 200 mL PBS) were subcutaneously injected into the right hind flank of three groups of mice (*n* = 6). After the tumor volume reached approximately 75 mm^3^, the mice were randomly divided into three groups and treated with vehicle or 10 mg/kg CTD. CTD was administered intraperitoneally once every two days over a 3-week window. Tumor size and body weight was recorded over the course of the treatment. Tumor volume was calculated using the formula: Volume = length × width × depth × 0.52. After the mice were euthanized, the tumors were extracted and weighed. The tumors were then either frozen in liquid nitrogen or fixed in 10% formalin and embedded in paraffin.

### 4.14. Statistical Analysis

Student’s *t*-test was used to perform statistical analysis for single comparisons. *p* < 0.05 was used as the criterion for statistical significance, and data are expressed as mean ± standard deviation.

## Data Availability

The datasets generated for this study are available on request to the corresponding author, without undue reservation.
